# A cumulative prospect theory-based method for group medical emergency decision-making with interval uncertainty

**DOI:** 10.1186/s12911-022-01867-w

**Published:** 2022-05-06

**Authors:** Jiayi Sun, Xiang Zhou, Juan Zhang, Kemei Xiang, Xiaoxiong Zhang, Ling Li

**Affiliations:** 1 School of Health Sciences, Jiangsu Food & Pharmaceutical Science College, Huai’an, Jiangsu China; 2 Department of Rehabilitation, Huai’an Second People’s Hospital, The Affiliated Huai’an Hospital of Xuzhou Medical University, Huai’an, Jiangsu China; 3 The Sixty-Third Research Institute, National University of Defense Technology, Nanjing, Jiangsu China; 4 Department of Dermatological, Lianshui County People’s Hospital, Affiliated Hospital of Kangda College, Nanjing Medical University, Huai’an, Jiangsu China

**Keywords:** Cumulative prospect theory, Group medical decision-making, Multi-attribute model, Interval value

## Abstract

**Background:**

An emergency response to a medical situation is generally considered to be a risk decision-making problem. When an emergency event occurs, it makes sense to take into account more than one decision maker’s opinions and psychological behaviors. The existing research tends to ignore these multidimensional aspects. To fill this literature gap, we propose a multi-attribute model.

**Methods:**

The model is based on cumulative prospect theory (CPT), considering multiple experts’ psychological factors. By not assuming full rationality, we extend existing models to allow multiple experts’ risk preferences to be incorporated into the decision-making process in the case of an emergency. Then, traditional CPT is extended by allowing for multiple attributes. In addition, rather than using crisp data, interval values are adopted to tackle the usual uncertainties in reality.

**Results:**

The multi-attribute CPT based model proposed can deal with the selection of potential emergency alternatives. The model incorporates interval values to allow more uncertainty and the comparative studies show that the optimal solution changes under different scenarios.

**Conclusions:**

Our illustrative example and comparative study show that considering multiple experts and multiple attributes is more reasonable, especially in complicated situations under an emergency. In addition, decision-makers’ risk preferences highly affect the selection outcomes, highlighting their importance in the medical decision-making process. Our proposed model can be applied to similar fields with appropriate modifications.

## Background

Emergency events, which require a reasonable and desirable response action, have drawn great attention recently. In general, decision-making problems in these situations are complicated by many factors and large volumes of data [1]. For example, in the medical field, physicians often face difficult decisions choosing from various alternatives in an emergency. Is a new surgical procedure safer than the old one? Which therapy is the most efficient for treating a malignancy? Medical decision-making processes can be complex, dynamic, and affected by time pressure, especially in emergency departments [2]. Unscientific decisions are likely to result in waste of resources or may negatively impact human life and social development. As a result, making the appropriate choice is vital, especially under uncertainty and lack of information [3, 4].

Various decision analysis methods have been investigated to address emergency response problems. In summary, the common methods used to tackle this issue can be classified into: multi-criteria decision-making methods, mathematical programming methods, and intelligence methods. These will be discussed in the next section. Despite significant contributions to the emergency decision-making problem in various fields, some weaknesses need to be addressed. First, decision makers (DMs) tend to deviate, especially when dealing with uncertainties. For instance, a DM is more likely to be risk averse when facing gains with certainty. On the contrary, he (she) is more prone to be risk-seeking when facing losses with probabilities [5]. Second, DMs may experience difficulty assigning crisp values to alternatives owing to certain factors in many real-world applications, like time pressure, or limited knowledge. Thus, it makes more sense to use interval values to express uncertainty, which is more practical than using numerical data [6].

Current research in medical emergency decision-making has focused on the impact of emergency events only on a single attribute basis. However, the benefit of a successful medical emergency event depends on more than one aspect, including cost and treatment effect, to name a few. Furthermore, more than one physician may be needed to manage a medical emergency problem effectively, especially under a complex and uncertain decision environment. Not only does the physician require knowledge about the underlying disease process and the most current treatment options available, but many other variables also need to be understood. Consequently, multiple experts and attributes need to be taken into account in the medical health domain [7]. In this regard, a more accurate medical decision-making model is needed to describe DMs’ behaviors, especially under a fuzzy environment.

To address the above-mentioned problems in the existing research, this study proposes a multi-attribute cumulative prospect theory (CPT)-based model. The main contributions of this paper are as follows.

1. Instead of assuming full rationality, this model allows multiple medical paramedics to incorporate individuals’ psychological preferences into the decision-making process in the case of an emergency.
2. Traditional CPT is extended by allowing for multiple attributes to cope with complex environments, especially through the emergency decision making process.
3. Rather than using crisp data, this study uses interval values to measure reference points and criteria values. Note that there are other studies using interval values as the reference points [8, 9] but few studies have incorporated the usage of interval values in medical emergency making with multiple attributes.

## Related works

Over the past few decades, emergency decision-making method in various fields has assumed great importance. In general, emergency decision-making is characterized by the pressure of little time and lack of information. For example, Ju et al. [10] studied a framework combining several multi-criteria decision-making methods to tackle the emergency alternative evaluation and selection problem. Zhou et al. [11] provided an overview of the emergency decision-making theory and methods for natural disasters in terms of a methodological perspective. Wan et al. [12] developed an interactive multi-criteria group decision-making method with probabilistic linguistic information and applied this method for emergency assistance for COVID-19 in Wuhan. In the medical decision-making field, Hazen et al. [13] introduced a stochastic tree model allowing for the explicit depiction of temporal uncertainty and applied this model to a medical scenario. Shea and Hoyt [14] discussed the role of nurse practitioners or physician assistants in an emergency or urgent-care setting and the necessary components leading to a sound medical decision-making process. Liao et al. [15] applied artificial intelligence to aid nurses in addressing problems and receiving instructions through information technology.

DMs are hardly rational in reality, Tversky and Kahneman [16] were the first to analyze Prospect Theory (PT) and Cumulative Prospect Theory (CPT) to better describe the decision behaviors of an individual under risk. Thanks to its simple logic and computation, this theory has also been applied in emergency decision-making. For example, Zhang et al. [17] proposed an approach based on PT considering experts’ psychological behavior and different emergency situations. Wang et al. [8] developed a PT-based interval dynamic reference point method for emergency decision-making. Liu et al. [18] proposed a hybrid method combining CPT and Choquet integral method to solve the risk decision-making problem in emergency response.

In addition, emergency decision making often involves a group of DMs. In general, experts or DMs with different background knowledge hold different or even controversial opinions. Therefore, different opinions need to be considered to reach a consensus and obtain a collective preference before the final solution. Following this study, Sun et al. [19] proposed a theoretical framework for a dynamic feedback mechanism in group decision making (GDM) using an attitudinal consensus threshold to generate certain recommendation advice for experts. Wu et al. [20] studied the influence of the group attitude on the consensus reaching process in GDM. Zhang and Li [21] developed some personalized individual semantics based consistency control and consensus reaching models for linguistic GDM. Wang et al. [9] developed a new group emergency decision making method considering experts’ psychological behaviors. Xu et al. [22] proposed a two-stage risk emergency decision-making method considering large groups based on social media big data; a real case study associated with the Tianjin port explosion on August 12, 2015, demonstrates the feasibility and effectiveness of this model. Wan et al. [23] proposed a new personalized a personalized individual semantics based consensus reaching process for large-scale GDM with probabilistic linguistic preference relations, and applied this method to COVID-19 surveillance plans selection.

In summary, multiple methods have been proposed to deal with emergency decision-making problems. However, few studies have incorporated DMs’ physiological factors in the medical emergency decision-making field, especially with multiple experts and a fuzzy environment.

### Group medical emergency decision-making procedure

In this section, we discuss briefly the notations and procedure of the group medical emergency decision-making problem.

### Notations

The notations used in the model formation are explained in Table 1.

**Table 1:** Notations used in this study

*Index*	
*h*	Index of experts
*m*	Index of response actions (alternatives) (*m* = 1, 2, …, *M*)
*n*	Index of outcomes (*n* = 1, 2, …, *N*)
*k*	Index of attributes (*k* = 1, 2, …, *K*)
*Parameter*	
*H*	Number of experts
*M*	Number of response actions (alternatives)
*N*	Number of possible outcomes in terms of different responses
*K*	Number of attributes considered in the medical emergency problem
*E*_*hk*_	Reference point value of expert *E*_*h*_ regarding criterion *k*
*R*_*hk*_	Normalized reference point value of expert *E*_*h*_ regarding criterion *k*
$\overline{R}_{k}$	Mean reference point value regarding criterion *k*
*r*_*k*_	Collective reference point value regarding criterion *k*
*x*_*mnk*_	*k*th attribute value with respect to the *n*th outcome of the *m*th action
*z*_*mnk*_	Corresponding gain or loss regarding each value
*v*_*mnk*_	Normalized gain or loss regarding each value
*Set*	
*E*	Group of experts
*A*	Set of all feasible response actions in a medical emergency
*P*	Vector of probabilities with respect to various outcomes

### Solution procedure

**Fig. 1:**
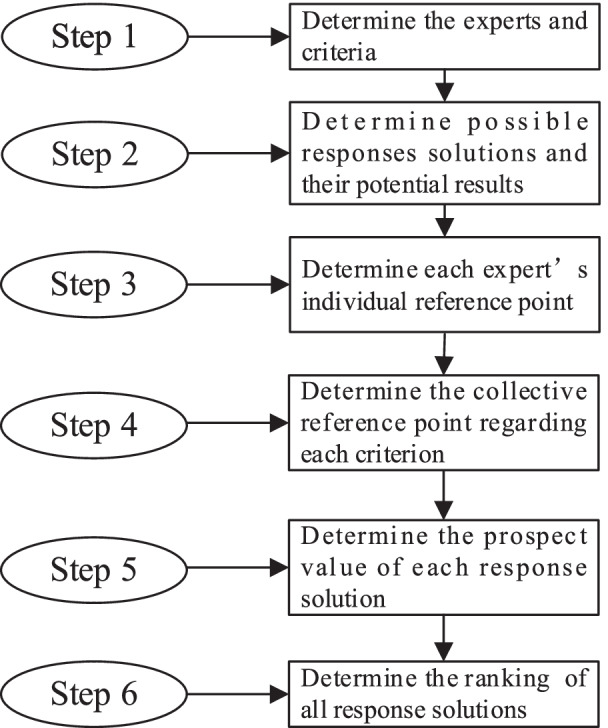
Solution procedure for the group medical emergency decision-making problem

To solve the group medical decision-making problem, a corresponding procedure is analyzed, as illustrated in Fig. 1. In step 1, a group of experts (physicians) and the criteria are determined. Then, in step 2, given a set of feasible emergency responses, we determine the value of all the potential responses for each criterion. In step 3, different experts are asked to express their unique reference points for each criterion. Interval values are used to allow for more uncertainty concerning the reference points and criterion values. To obtain a collective reference point, these experts are assigned weights based directly on their opinions. In step 4, a collective reference point is constructed combining all the experts’ opinions using their weights. Next, the relative gains and losses of each solution outcomes on each criterion are calculated based on CPT. In step 5, the prospect values of different response alternatives are obtained, based on which the rankings of all response alternatives can be determined.

**Fig. 2:**
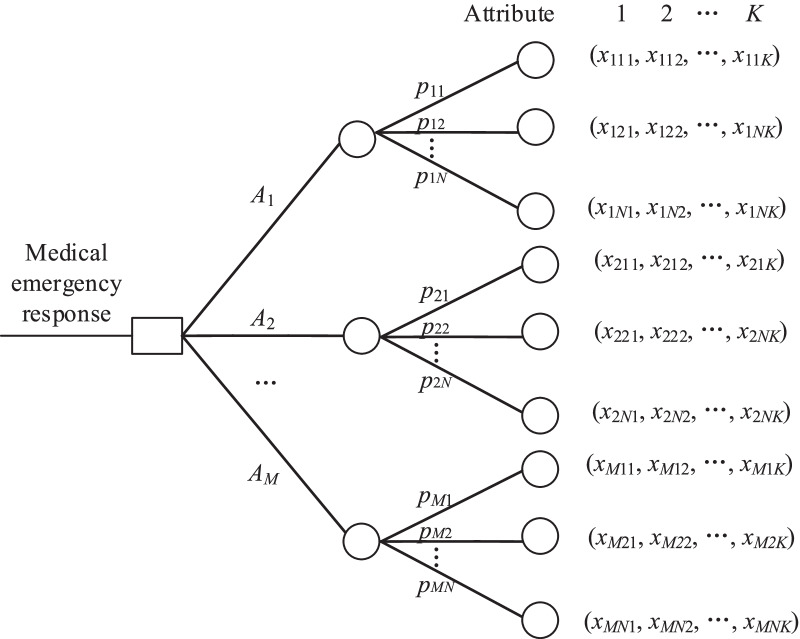
Description of risk decision-making in a medical emergency response

Experts are required to select a desirable response solution among a set of different alternatives in a specific medical emergency situation. As depicted in step 5 in Fig. 1, the prospect value of each response alternative needs to be determined. Figure 2 depicts a brief illustration of the problem [18]. $A_{m} {\kern 1pt} {\kern 1pt} (m = 1,2,..,M)$ refers to different emergency response solutions. $p_{n} {\kern 1pt} {\kern 1pt} (n = 1,2,..,N)$ denotes the possibility of each outcome under the corresponding solution. *x*_*mnk*_ refers to the *k*th attribute value with respect to the *n*th outcome $(m = 1,..,M;{\kern 1pt} {\kern 1pt} n = 1,..,N;{\kern 1pt} {\kern 1pt} {\kern 1pt} k = 1,..,K)$ of the *m*th solution. Let *r*_*k*_ denote the collective *k*th reference point with respect to attribute *k*, separating losses from gains. The determination of the collective reference point value *r*_*k*_ considering each expert’s preferences is briefly introduced in the “Basic model” section. Due to the complexities of such an emergency decision-making problem, we use interval values rather than crisp values to measure these parameters, including *x*_*mnk*_ and *r*_*k*_.

## Methods

This section investigates in detail the proposed emergency decision-making model based on CPT with interval uncertainty consideration. In the “Information gathering” section, we briefly introduce the calculation of a collective reference point for each criterion. The “Payoff calculation” section presents how the payoffs with interval uncertainty are obtained. Finally, we explore the ranking order of different emergency response solutions in terms of their prospect values.

### Information gathering

As mentioned in the Introduction, individuals tend to treat outcomes as gains and losses from reference points. Therefore, the values of reference points are vital throughout the decision-making process. In existing studies, the reference points are generally based on crisp values. Uncertainty, however, exists in most real-world cases, calling for a more flexible model for a better measure of reality. To address this issue, we introduce interval values to represent reference points and attribute values in this study. Note that in GDM, each individual may have different reference points for each criterion, and therefore, it makes sense to incorporate all individuals’ opinions and obtain a collective reference point for each criterion.

There are multiple methods to aggregate the DM’s preferences, such as the weighted sum method [24], OWA [25], and IOWA [26]. However, this problem is neither a major concern in this paper, nor a key component of our model. Therefore, the most commonly used method, i.e., the weighted sum method, is adopted in this study to combine each individual’s preference value. The expert’s weight, however, is not given in advance. To address this issue, we follow Chen and Yang [27]—the closer an expert’s preference value is to the mean value, the larger should be the assigned weight.

Following tradition, let {*E*_1_, *E*_2_, …, *E*_*H*_} be a group of experts characterized by their unique backgrounds and knowledge, and *K* criteria are considered in the group emergency decision-making problem. Assume that a decision matrix with interval numbers is formulated as

1 $$\begin{array}{*{20}c} {} & {Criterion{\kern 1pt} {\kern 1pt} 1} & {Criterion{\kern 1pt} {\kern 1pt} 2} & \cdots & {Criterion{\kern 1pt} {\kern 1pt} K} \\ {E_{1} } & {\left[ {E_{11}^{L} ,E_{11}^{H} } \right]} & {\left[ {E_{12}^{L} ,E_{12}^{H} } \right]} & \cdots & {\left[ {E_{1K}^{L} ,E_{1K}^{H} } \right]} \\ {E_{2} } & {\left[ {E_{21}^{L} ,E_{21}^{H} } \right]} & {\left[ {E_{22}^{L} ,E_{22}^{H} } \right]} & \cdots & {\left[ {E_{2K}^{L} ,E_{2K}^{H} } \right]} \\ \cdots & \cdots & \cdots & \cdots & \cdots \\ {E_{H} } & {\left[ {E_{H1}^{L} ,R_{H1}^{H} } \right]} & {\left[ {E_{H2}^{L} ,E_{H2}^{H} } \right]} & \cdots & {\left[ {E_{HK}^{L} ,E_{HK}^{H} } \right]} \\ \end{array} ,$$

where *E*_*hk*_ stands for the reference point value of expert *E*_*h*_ regarding criterion *k*, denoted as an interval number $\left[ {E_{hk}^{L} ,E_{hk}^{H} } \right]$.

In particular, we need to, first, normalize the matrix so that the reference point value regarding different criteria can be normalized later. For that purpose, each *E*_*hk*_ is normalized to *R*_*hk*_ using the following relation for the benefit-type criterion:

2 $$R_{hk}^{L} = \frac{{E_{hk}^{L} - \min_{h} E_{hk} }}{{\max_{h} E_{hk} - \min_{h} E_{hk} }},\;\;\;R_{hk}^{H} = \frac{{E_{hk}^{H} - \min_{h} E_{hk} }}{{\max_{h} E_{hk} - \min_{h} E_{hk} }}$$

For the cost-type criterion, the following relation is adopted as

3 $$R_{hk}^{L} = \frac{{\max_{h} E_{hk} - E_{hk}^{L} }}{{\max_{h} E_{hk} - \min_{h} E_{hk} }},\;\;\;{\kern 1pt} {\kern 1pt} R_{hk}^{H} = \frac{{\max_{h} E_{hk} - E_{hk}^{H} }}{{\max_{h} E_{hk} - \min_{h} E_{hk} }}$$

where $\max_{h} E_{hk} = \max (\max_{h} E_{hk}^{L} ,\max_{h} E_{hk}^{H} ),{\kern 1pt} {\kern 1pt} \min_{h} E_{hk} = \min (\min_{h} E_{hk}^{L} ,\min_{h} E_{hk}^{H} )$. The normalized *R*_*hk*_ has an apparent value between 0 and 1.

Then, the normalized decision-matrix describing experts’ preferences on the reference point is

$$\begin{array}{*{20}c} {} & {Criterion{\kern 1pt} {\kern 1pt} 1} & {Criterion{\kern 1pt} {\kern 1pt} 2} & \cdots & {Criterion{\kern 1pt} {\kern 1pt} K} \\ {E_{1} } & {\left[ {R_{11}^{L} ,R_{11}^{H} } \right]} & {\left[ {R_{12}^{L} ,R_{12}^{H} } \right]} & \cdots & {\left[ {R_{1K}^{L} ,R_{1K}^{H} } \right]} \\ {E_{2} } & {\left[ {R_{21}^{L} ,R_{21}^{H} } \right]} & {\left[ {R_{22}^{L} ,R_{22}^{H} } \right]} & \cdots & {\left[ {R_{2K}^{L} ,R_{2K}^{H} } \right]} \\ \cdots & \cdots & \cdots & \cdots & \cdots \\ {E_{H} } & {\left[ {R_{H1}^{L} ,R_{H1}^{H} } \right]} & {\left[ {R_{H2}^{L} ,R_{H2}^{H} } \right]} & \cdots & {\left[ {R_{HK}^{L} ,R_{HK}^{H} } \right]} \\ \end{array}$$

We next obtain the mean reference point value $\overline{R}_{k} = [\overline{R}_{{_{k} }}^{L} ,\overline{R}_{{_{k} }}^{H} ]$ for all experts for each criterion, calculated as follows:

4 $$\overline{R}_{k}^{L} = \frac{1}{H}\sum\limits_{h = 1}^{H} {R_{hk}^{L} } ,{\kern 1pt} \;\;\;{\kern 1pt} \overline{R}_{k}^{H} = \frac{1}{H}\sum\limits_{h = 1}^{H} {R_{hk}^{H} }$$

Note that $$\overline{R}_{k} = [\overline{R}_{{_{k} }}^{L} ,\overline{R}_{{_{k} }}^{H} ]$$.

Then, the distance between the preference of expert *h*, that is, $R_{hk} = \left[ {E_{hk}^{L} ,E_{hk}^{H} } \right]$, and the mean reference point regarding criterion *k*, $\overline{R}_{k} = [\overline{R}_{{_{k} }}^{L} ,\overline{R}_{{_{k} }}^{H} ]$, is obtained as

5 $$d_{hk} = \sqrt {\frac{{(E_{hk}^{L} - \overline{R}_{{_{k} }}^{L} )^{2} + (E_{hk}^{H} - \overline{R}_{{_{k} }}^{H} )^{2} }}{2}} ,$$

Thereafter, a distance matrix *D* with numerical number is formulated as

6 $$\begin{array}{*{20}c} {} & {Criterion{\kern 1pt} {\kern 1pt} 1} & {Criterion{\kern 1pt} {\kern 1pt} 2} & \cdots & {Criterion{\kern 1pt} {\kern 1pt} K} \\ {E_{1} } & {d_{11} } & {d_{12} } & \cdots & {d_{K1} } \\ {E_{2} } & {d_{21} } & {d_{22} } & \cdots & {d_{K2} } \\ \cdots & \cdots & \cdots & \cdots & \cdots \\ {E_{H} } & {d_{H1} } & {d_{H2} } & \cdots & {d_{HK} } \\ \end{array}$$

The similarity between each expert’s opinions and the mean value is measured using

7 $$d_{h} = \sum\limits_{k = 1}^{K} {(1 - d_{hk} )}$$

As mentioned earlier, an expert with a more similar value to the mean value is given a greater weight, computed as

8 $$w_{h} = d_{h} /\sum\limits_{h = 1}^{H} {d_{h} } .$$

When the weights have been obtained, we then combine all the experts’ opinions into a global one. The collective reference point for criterion *k* is computed as

9 $$r_{k} = \sum\limits_{h = 1}^{H} {w_{h} \times R_{hk} } {\kern 1pt} \Rightarrow \left[ {r_{k}^{L} ,r_{k}^{U} } \right] = \sum\limits_{h = 1}^{H} {w_{h} \times \left[ {R_{hk}^{L} ,R_{hk}^{U} } \right]}$$

### Payoff calculation

Suppose that $R = [R^{L} ,R^{H} ]$ and $C = [C^{L} ,C^{H} ]$ are two interval values. In particular, let $R = [R^{L} ,R^{H} ]$ represent the reference point, dividing gains and losses regarding different outcomes. Table 2 shows the possible relationships between these two reference points [8].

**Table 2:**
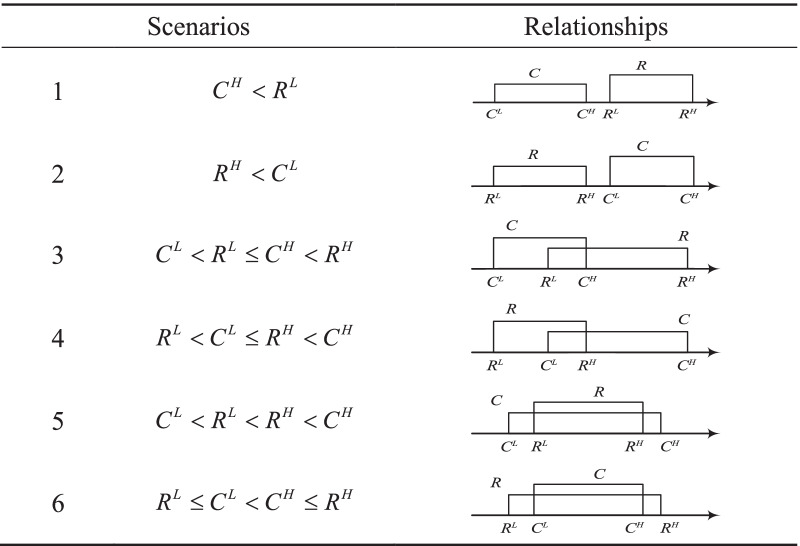
Six possible relationships between *R* and *C*

In other words, for the benefit type criteria, an attribute value greater than the reference value is treated as a gain to the individual, but for the cost type (e.g., time, cost), the smaller the value, the better. The attribute value is treated as a gain only if it is less than the reference value; otherwise, it is a loss.

To calculate the relationship between two interval values, we first form the following definition. Let $C = [C^{L} ,C^{H} ]$ be an interval attribute value, and *x* be a random variable with uniform distribution. Then, the probability density function of *x* is expressed using the following form

10 $$f(x) = \left\{ {\begin{array}{*{20}l} {\frac{1}{{C^{H} - C^{L} }},} \hfill & {C^{L} \le x \le C^{H} } \hfill \\ {0,} \hfill & {otherwise} \hfill \\ \end{array} ,} \right.$$

where $$\int_{{C^{L} }}^{{C^{H} }} {f(x)dx = 1}$$.

As there are six possible relationships between two interval values, we take only the first case in Table 2 to illustrate how the payoff for the individual is obtained. Note that the following calculation is for the benefit criterion. Obviously, no gain can be obtained, as $C_{{}}^{H} < R_{{}}^{L}$. The loss is given by

11 $$\begin{aligned} L & = \int_{{C^{L} }}^{{C^{H} }} {(x - R^{L} )f(x)dx} \\ & \quad = 0.5 * (C^{L} + C^{H} ) - R^{L} \\ \end{aligned}$$

Refer to Wang et al. [8] for more details. In summary, the payoffs for all possible scenarios under the two different criteria types are tabulated in Table 3.

**Table 3:** Payoffs for the benefit type criteria in six scenarios

Case	Relation	Benefit type		Cost type	
		Loss	Gain	Loss	Gain
1	$C_{{}}^{H} < R_{{}}^{L}$	$0.5(C_{{}}^{L} + C_{{}}^{H} ) - R_{{}}^{L}$	0	$R_{{}}^{L} - 0.5(C_{{}}^{L} + C_{{}}^{H} )$	0
2	$R_{{}}^{H} < C_{{}}^{L}$	0	$0.5(C_{{}}^{L} + C_{{}}^{H} ) - R_{{}}^{H}$	0	$R_{{}}^{H} - 0.5(C_{{}}^{L} + C_{{}}^{H} )$
3	$C_{{}}^{L} < R_{{}}^{L} \le C_{{}}^{H} < R_{{}}^{H}$	$0.5(C_{{}}^{L} - R_{{}}^{L} )$	0	$0.5(R_{{}}^{L} - C_{{}}^{L} )$	0
4	$R_{{}}^{L} < C_{{}}^{L} \le R_{{}}^{H} < C_{{}}^{H}$	0	$0.5(C_{{}}^{H} - R_{{}}^{H} )$	0	$0.5(R_{{}}^{H} - C_{{}}^{H} )$
5	$C_{{}}^{L} < R_{{}}^{L} < R_{{}}^{H} < C_{{}}^{H}$	$0.5(C_{{}}^{L} - R_{{}}^{L} )$	$0.5(C_{{}}^{H} - R_{{}}^{H} )$	$0.5(R_{{}}^{L} - C_{{}}^{L} )$	$0.5(R_{{}}^{H} - C_{{}}^{H} )$
6	$R_{{}}^{L} \le C_{{}}^{L} < C_{{}}^{H} \le R_{{}}^{H}$	0	0	0	0

### Prospect value calculation

This subsection explores how the prospect value is obtained based on CPT. Assume that *M* possible solutions exist in the case of a medical emergency. *N* different outcomes may occur under each solution with certain possibilities, resulting in various consequences on *K* criteria. We aim to determine the optimal solution for the experts based on their preferences.

To calculate the prospect value of each response alternative, we first need to measure its outcome on each criterion. As described before, more than one criterion is allowed in this study. According to the collective reference point *r*_*k*_ and the interval attribute value *x*_*mnk*_, the gain or loss to the DM, denoted as *z*_*mnk*_, can be obtained using Table 3 based on the criterion type. Then, for each *z*_*mnk*_, the following PT function is applied to measure its outcome on each criterion

12 $$v_{mnk} = \left\{ {\begin{array}{*{20}l} {z_{mnk}^{\alpha } ,} \hfill & {z_{mnk} \ge 0} \hfill \\ { - \lambda ( - z_{mnk} )^{\beta } ,} \hfill & {z_{mnk} < 0} \hfill \\ \end{array} } \right..$$

where *v*_*mnk*_ refers to the normalized gain or loss for each criterion value.

Note that as more than one attribute is considered, different outcomes on each attribute need to be normalized before aggregation [28]. Therefore, the following process is carried out to normalize each attribute value as

13 $$\tilde{v}_{mnk} = \frac{{v_{mnk} }}{{v_{k}^{*} }},\;\;\;\;{\kern 1pt} m = 1,2, \ldots ,M;{\kern 1pt} \;\;\;n = 1,2, \ldots ,N;{\kern 1pt} \;\;\;k = 1,2, \ldots ,K,$$

where $$v_{k}^{*} = \max_{{m \in M,{\kern 1pt} {\kern 1pt} n \in N}} \{ |v_{mnk} |\}$$.

The weighting function in CPT is another important factor that cannot be overlooked. For the sake of computation, we adopt a neo-additive probability weighting function [29], shown as

14 $$w(p) = \left\{ {\begin{array}{*{20}l} 1 \hfill & {p = 1} \hfill \\ {\mu p + \frac{1}{2}(1 - \mu )} \hfill & {0 < p < 1} \hfill \\ 0 \hfill & {p = 0} \hfill \\ \end{array} } \right.,$$

where $0 < \mu < 1$. When *μ* is equal to 1, Eq. (14) can read as $w(p) = p$, representing no probability weighting. This simple formulation of the function provides the essential features of CPT in that small probabilities tend to be overweighted whereas big probabilities tend to be underweighted. Note that researchers have recommended different values under certain situations in terms of *μ.* However, such a problem is neither the major concern nor a key component of our study. We set *μ* as 0.6 in this study.

After we obtain the normalized criterion value and weighting function, the prospect value of each event is obtained using the formulation of Bleichrodt et al. [30]. The normalized outcomes $\tilde{v}_{mnk}$ (${\kern 1pt} m = 1,2,...,M;{\kern 1pt} {\kern 1pt} {\kern 1pt} n = 1,2,...,N;{\kern 1pt} {\kern 1pt} {\kern 1pt} k = 1,2,...,K$) are ranked in descending order as $\tilde{v}_{mnk} \ge \tilde{v}_{m,n + 1,k}$. Outcome $\tilde{v}_{mnk}$ occurs with probability $p_{ik}$, and attribute *j* has *m*_*i*_ different outcomes that are gains regarding prospect *i*. Next, the prospect value of the *i*th event solution under this condition is formulated as

15 $$\begin{aligned} PV_{i} & = w_{1} \left( {\sum\limits_{k = 1}^{{m_{1} }} {\pi_{1}^{ + } (p_{ik} )\tilde{v}_{ik1} + } \sum\limits_{{k = m_{1} { + }1}}^{n} {\pi_{1}^{ - } (p_{ik} )\tilde{v}_{ik1} } } \right) \\ & \quad + w_{2} \left( {\sum\limits_{k = 1}^{{m_{2} }} {\pi_{2}^{ + } (p_{ik} )\tilde{v}_{ik2} + } \sum\limits_{{k = m_{2} { + }1}}^{n} {\pi_{2}^{ - } (p_{ik} )\tilde{v}_{ik2} } } \right) \\ & \quad + {\kern 1pt} \ldots \\ & \quad w_{J} \left( {\sum\limits_{k = 1}^{{m_{J} }} {\pi_{J}^{ + } (p_{ik} )\tilde{v}_{ikJ} + } \sum\limits_{{k = m_{J} { + }1}}^{n} {\pi_{J}^{ - } (p_{ik} )\tilde{v}_{ikJ} } } \right), \\ \end{aligned}$$

where $\pi_{j}^{ + } (p_{ik} ) = w_{j}^{ + } (p_{i1} ,{\kern 1pt} {\kern 1pt} ...,{\kern 1pt} {\kern 1pt} p_{ik} ) - w_{j}^{ + } (p_{i1} ,{\kern 1pt} {\kern 1pt} ...,{\kern 1pt} {\kern 1pt} p_{{i,{\kern 1pt} {\kern 1pt} k - 1}} )$ and $\pi_{j}^{ - } (p_{ik} ) = w_{j}^{ - } (p_{ik} ,{\kern 1pt} {\kern 1pt} ...,{\kern 1pt} {\kern 1pt} p_{in} ) - w_{j}^{ - } (p_{{i,{\kern 1pt} k + 1}} ,{\kern 1pt} {\kern 1pt} ...,{\kern 1pt} {\kern 1pt} p_{in} )$. $\pi_{j}^{ + } (p)$ and $\pi_{j}^{ - } (p)$ are the decision weights for the *j*th attribute’s gains and losses, respectively. $\tilde{v}_{ikj}$ is the normalized value obtained in Eq. (13). *w*_1_, *w*_2_, …, *w*_*J*_ are attribute weights summing to one.

## Results

This section presents an example to illustrate the feasibility and applicability of the proposed model and procedure when dealing with medical emergency situations. In addition, we carry out comparative studies to observe how the parameter values affect the results.

### Basic model

Assume an emergency scenario of an individual involved in an accident, who needs immediate surgery. Based on the symptoms and analysis by four experts, three feasible actions are proposed for how to deal with the emergency:

- *A*_1_: traditional method
- *A*_2_: standard treatment method
- *A*_3_: new therapy method

To select a desirable response action to the emergency, three criteria are considered:

- *C*_1_: main immediate treatment effect
- *C*_2_: potential effects after surgery in the long run
- *C*_3_: positive ripple effect on the hospital

Criteria *C*_1_, *C*_2_, and *C*_3_ are the benefit types. In other words, a greater value is preferred. Due to the complexities of the problem, DMs would rather use crisp values representing the reference points. The weights for the three criteria are 0.4, 0.35, and 0.25. The scale parameter is set as 1 and the loss aversion parameter is $\lambda = 2$.

Basically, the surgery can either succeed or fail for each response action. Thus, only two situations can occur for each solution. We assume that the success and failure possibilities for each solution are *p*_11_ = 0.75, *p*_12_ = 0.25, *p*_21_ = 0.8, *p*_22_ = 0.2, *p*_31_ = 0.7, and *p*_32_ = 0.3. We argue that the parameter settings and values are for illustrative purposes only. Such a problem is neither the major concern in our study, nor a key factor for our emergency decision-making model. In practice, however, these values can be determined by expert elicitation, historical statistics, or experiments [28].

To tackle this emergency decision-making problem, the proposed procedure is adopted. The solution process is explained step by step below. First, the criteria, reference points, and possible emergency response are determined as mentioned above. Then, four experts (physicians) give their individual reference point value for each criterion using interval values, denoted as

$$\begin{array}{*{20}c} {} & {Criterion{\kern 1pt} {\kern 1pt} 1} & {Criterion{\kern 1pt} {\kern 1pt} 2} & {Criterion{\kern 1pt} {\kern 1pt} 3} & {} \\ {E_{1} } & {\left[ {40,48} \right]} & {\left[ {50,55} \right]} & {\left[ {0.48,0.58} \right]} & {} \\ {E_{2} } & {\left[ {38,46} \right]} & {\left[ {48,54} \right]} & {\left[ {0.45,0.54} \right]} & {} \\ {E_{3} } & {\left[ {45,50} \right]} & {\left[ {52,56} \right]} & {\left[ {0.50,0.60} \right]} & {} \\ {E_{4} } & {\left[ {48,52} \right]} & {\left[ {50,54} \right]} & {\left[ {0.55,0.62} \right]} & {} \\ \end{array}$$

To begin with, we normalize matrix *E* for the reference point value using Eqs. (2)–(3), obtained as

$$\begin{array}{*{20}c} {} & {Criterion{\kern 1pt} {\kern 1pt} 1} & {Criterion{\kern 1pt} {\kern 1pt} 2} & {Criterion{\kern 1pt} {\kern 1pt} 3} & {} \\ {E_{1} } & {\left[ {0.143,0.714} \right]} & {[0.25,0.875]} & {\left[ {0.177,0.765} \right]} & {} \\ {E_{2} } & {[0,0.571]} & {\left[ {0,0.75} \right]} & {\left[ {0,0.529} \right]} & {} \\ {E_{3} } & {\left[ {0.5,0.857} \right]} & {[0.5,1.0]} & {\left[ {0.294,0.882} \right]} & {} \\ {E_{4} } & {\left[ {0.714,1.0} \right]} & {\left[ {0.25,0.75} \right]} & {\left[ {0.588,1.0} \right]} & {} \\ \end{array}$$

The mean reference point for each criterion is obtained as $\overline{R}_{1} = [0.339,0.786],\overline{R}_{2} = [0.25,0.844],\overline{R}_{3} = [0.265,0.794]$. Then, the distance matrix between each expert and the mean preference value is calculated using Eq. (5) as

$$\begin{array}{*{20}c} {0.148} & {0.022} & {0.066} \\ {0.284} & {0.189} & {0.265} \\ {0.124} & {0.209} & {0.066} \\ {0.305} & {0.066} & {0.271} \\ \end{array}$$

Take *d*_11_ in the above matrix as an example. It is calculated as follows:

$$d_{11} = \sqrt {\frac{{(0.143 - 0.339)^{2} + (0.714 - 0.786)^{2} }}{2}} = 0.148$$

Next, the weights of the experts are obtained using Eqs. (7)–(8) as $w_{1} = 0.277,w_{2} = 0.227,w_{3} = 0.261,w_{4} = 0.236$.

Last, a collective reference point can be calculated using the weighted sum method as $r_{1} = [42.738,49.012],r_{2} = [50.068,54.798],r_{3} = [0.495,0.586]$.

For step 5 in Fig. 1, the possible outcomes of each solution for each criterion are collected and tabulated in Table 4.

**Table 4:** Outcomes of three decision actions for each criterion

Solution	Succeed			Fail		
	*C*_1_	*C*_2_	*C*_3_	*C*_1_	*C*_2_	*C*_3_
*A*_1_	[60, 70]	[80, 85]	[0.75, 0.8]	[30, 40]	[40, 45]	[0.35, 0.4]
*A*_2_	[70, 75]	[72, 80]	[0.75, 0.85]	[32, 42]	[46, 48]	[0.45, 0.5]
*A*_3_	[82, 88]	[75, 80]	[0.8, 0.84]	[36, 44]	[35, 40]	[0.48, 0.5]

Based on the procedure of the decision-making model, we first need to calculate the relative payoff of all solutions for each criterion. To solve this, the relative payoffs are tabulated in Table 5 (based on Table 3). Note that crisp values, instead of interval values, for each criterion are obtained.

**Table 5:** Relative payoffs of three decision actions for each criterion

Solution	Succeed			Fail		
	*C*_1_	*C*_2_	*C*_3_	*C*_1_	*C*_2_	*C*_3_
*A*_1_	15.988	27.702	0.189	− 7.738	− 7.568	− 0.120
*A*_2_	23.488	21.202	0.214	− 5.738	− 3.068	− 0.023
*A*_3_	35.988	22.702	0.234	− 3.369	− 12.568	− 0.008

Take the first item in Table 5 as an example. *C* is [60, 70] and *R* (i.e., *r*_1_) is [42.738, 49.012]. In this case, we have $R_{{}}^{H} < C_{{}}^{L}$ and the second scenario is satisfied. Therefore, the payoff under this case is $0.5(C^{L} + C^{H} ) - R^{H} = 0.5*(60 + 70) - 49.012 = 65 - 49.012 = 15.988$. The same logic applies in other situations too. First, Eq. (12) is used to compute the relative gains or losses on each criterion using CPT. Then, Eq. (13) is used to normalize each attribute value so that they can be aggregated. The weighting function is used to transfer the possibility value of each solution outcome using Eq. (14). Take the first transition weight as an example, $w(p_{11} ) = 0.6*0.75 + 0.5*(1 - 0.6) = 0.65$. Transition weights are the transferred weights (subjective probabilities) rather than the objective probabilities based on the weighting function in CPT. Table 6 gives the relatives payoffs and transition weights for each solution using CPT. Finally, we use Eq. (15) to calculate the prospect value of each solution.

**Table 6:** Prospect values of three decision actions

Solution	Normalized value		Transition weights		Prospect value
	Succeed	Fail	Succeed	Fail	
*A*_1_	0.725	− 0.613	0.65	0.35	0.257
*A*_2_	0.752	− 0.252	0.68	0.32	0.431
*A*_3_	0.931	− 0.408	0.62	0.38	0.422

As there are only two possible outcomes of each solution, Eq. (15) can be simplified. Take *A*_1_, as an example, its prospect value is calculated based on Eq. (15) as

$$\begin{aligned} PV_{1} & = w_{1} \times \pi_{1}^{ + } (p_{ik} )\tilde{v}_{ik1} + w_{2} \times \pi_{2}^{ - } (p_{ik} )\tilde{v}_{ik2} \\ & = 0.725 \times 0.65 - 0.613 \times 0.35 \\ & = 0.257 \\ \end{aligned}$$

The prospect value of each of the three solutions is obtained as 0.257, 0.431, and 0.422, respectively. It is easy to obtain the following results: *PV*_2_ > *PV*_3_ > *PV*_1_. Accordingly, the solution with the maximum prospect value, that is, *A*_2_, is chosen as the best solution in this situation.

### Comparative analysis

#### Impact of criteria

**Fig. 3:**
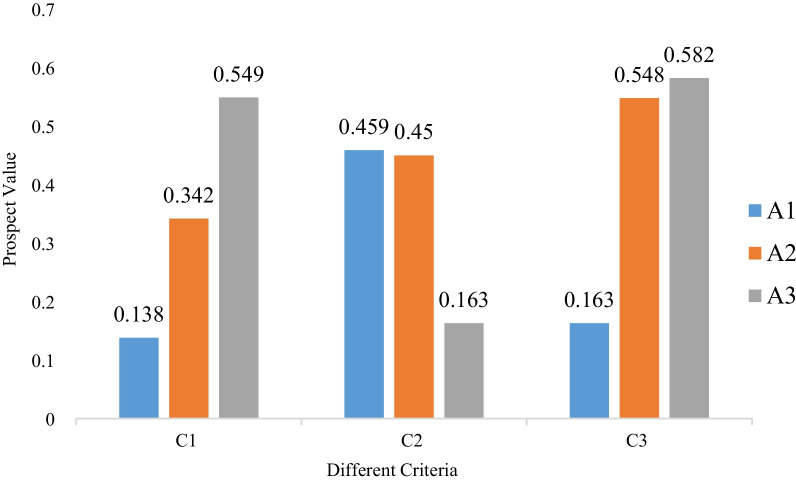
Prospect values of three solutions for each criterion

In this subsection, a comparative study taking into account only one attribute value is first carried out. Using the proposed method, we evaluate the prospect value of each solution considering each of the three attributes independently. Note that transition weights are adopted. Figure 3 gives the prospect values for three different solutions under each criterion and the corresponding ranking of alternatives with respect to each situation.

From Fig. 3, we find that the ranking order differs in three different situations. Specifically, if considering only criterion *C*_1_, the prospect value for each solution is 0.138, 0.342, and 0.549, respectively. Accordingly, the ranking order is *A*_3_, followed by *A*_2_ and *A*_1_. Considering criterion *C*_2_, the prospect value for each solution is 0.459, 0.450, and 0.163, respectively. For this reason, the optimal solution in this case is *A*_1_, followed by *A*_2_ and *A*_3_. When it comes to criterion *C*_3_, the ranking order is the same as that of criterion *C*_1_, although the prospect values differ.

#### Impact of outcome weights

For the sake of comparison, we continue the experiment with individuals having original weights, which refer to the objective probabilities as settled in the baseline. Table 7 gives the final outcomes. It is easy to obtain the following results: *PV*_3_ > *PV*_2_ > *PV*_1_. Accordingly, the solution having the maximum prospect value, that is, *A*_3_, is chosen as the best solution in this case. This outcome differs greatly from that in Table 6. The reason may be that small probabilities are overweighted, and large probabilities are underweighted when applying CPT in this model, leading to the change in prospect value of each solution. The change of the ranking order highlights, at least to an extent, the importance of considering transition weights throughout the decision-making process.

**Table 7:** Relative payoffs of three decision actions for each criterion under original weights

Solution	Normalized value		Original weights		Value
	Succeed	Fail	Succeed	Fail	
*A*_1_	0.730	− 0.310	0.75	0.25	0.470
*A*_2_	0.758	− 0.127	0.8	0.2	0.581
*A*_3_	0.937	− 0.204	0.7	0.3	0.594

#### Impact of experts’ weights

As multiple experts are considered in this study, all their opinions are taken into account. It is logical that different experts’ weights may impact the final solution. To address this issue, five scenarios are considered in Table 8. In particular, the first line shows the baseline, as illustrated in the “Basic model” section. For scenarios 1 to 4, only one expert’s opinions are considered. We aim to show the importance of GDM. In scenario 5, the four experts are set the same weights regardless of their opinions.

**Table 8:** Five different scenarios with respect to different experts’ weights

Scenario	Experts’ weights			
	*E*_1_	*E*_2_		*E*_3_
Baseline	0.277	0.227	0.261	0.236
1	1	0	0	0
2	0	1	0	0
3	0	0	1	0
4	0	0	0	1
5	0.25	0.25	0.25	0.25

**Fig. 4:**
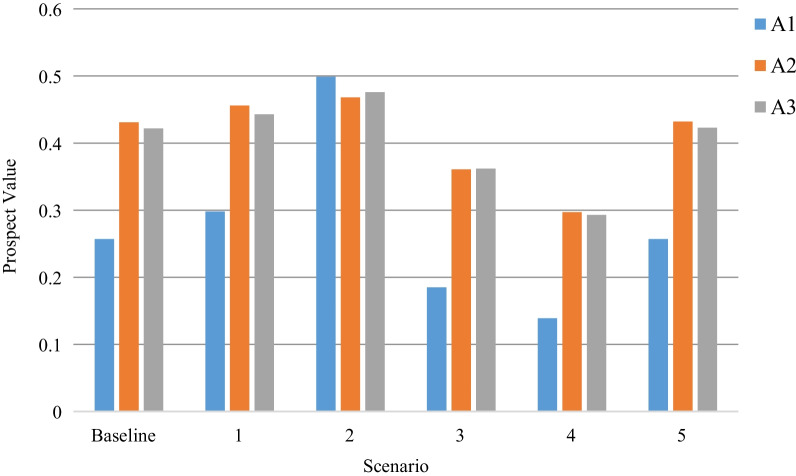
Prospect values of three solutions under different experts’ weights

From Fig. 4, the prospect values for each solution are different, and the optimal selection varies for different scenarios. In particular, the second solution, *A*_2_, is selected as the optimal solution for the baseline, scenario 1, scenario 4, and scenario 5. For scenario 2, in which only the second expert’s opinions are considered, solution *A*_1_ is chosen as the best one. For scenario 3, which considers only the third expert’s opinions, solution *A*_3_ is the optimal one. The differences highlight the importance of considering multiple experts’ opinions in decision-making, especially in the case of an emergency, where one DM might not be able to acquire all the important information.

## Conclusion

This study explores a group medical decision-making problem in the case of an emergency. Traditional methods seldom incorporate individuals’ risk preferences into decision-making, which is not realistic. In this study, a multi-attribute CPT based model is investigated to deal with the selection of potential emergency alternatives. We extend the existing research by incorporating interval values to allow more uncertainty in the model. Our illustrative example and comparative study show that considering multiple experts and multiple attributes is more reasonable, especially under complicated situations in an emergency. In addition, DMs’ risk preferences highly affect the selection outcomes, highlighting their importance in the medical decision-making process.

Several directions can be considered for future research. For example, more criteria could be considered, such as time or cost factors; other types of experts’ preferences can be adopted throughout decision making process, such as linguistic information or intuitionistic fuzzy preference relations. We aim to continue our research in this direction.

## Data Availability

All data generated or analysed during this study are included in this published article.

## Abbreviations

CPT: Cumulative prospect theory
GDM: Group decision-making
DM: Decision maker
